# Effect of EEG Electrode Numbers on Source Estimation in Motor Imagery

**DOI:** 10.3390/brainsci15070685

**Published:** 2025-06-26

**Authors:** Mustafa Yazıcı, Mustafa Ulutaş, Mukadder Okuyan

**Affiliations:** 1 Department of Computer Engineering, Faculty of Engineering, Karadeniz Technical University, Trabzon 61080, Türkiye; myazici@ktu.edu.tr (M.Y.); ulutas@ktu.edu.tr (M.U.); 2 Department of Physiology, Faculty of Medicine, Karadeniz Technical University, Trabzon 61080, Türkiye

**Keywords:** motor imagery, EEG, electrode number, brain–computer interface

## Abstract

The electroencephalogram (EEG) is one of the most popular neurophysiological methods in neuroscience. Scalp EEG measurements are obtained using various numbers of channels for both clinical and research applications. This pilot study explores the effect of EEG channel count on motor imagery classification using source analysis in brain–computer interface (BCI) applications. Different channel configurations are employed to evaluate classification performance. This study focuses on mu band signals, which are sensitive to motor imagery-related EEG changes. Common spatial patterns are utilized as a spatiotemporal filter to extract signal components relevant to the right hand and right foot extremities. Classification accuracies are obtained using configurations with 19, 30, 61, and 118 electrodes to determine the optimal number of electrodes in motor imagery studies. Experiments are conducted on the BCI Competition III Dataset Iva. The 19-channel configuration yields lower classification accuracy when compared to the others. The results from 118 channels are better than those from 19 channels but not as good as those from 30 and 61 channels. The best results are achieved when 61 channels are utilized. The average accuracy values are 83.63% with 19 channels, increasing to 84.70% with 30 channels, 84.73% with 61 channels, and decreasing to 83.95% when 118 channels are used.

## 1. Introduction

The brain is a complex and mysterious organ that has 100 billion connected neurons [1]. Many studies have been conducted to unravel the mystery of the brain, and it still has secrets. One of the popular types of research concerns brain–computer interfaces (BCIs) for people affected by paraplegia or quadriplegia due to cerebral palsy, stroke, amyotrophic lateral sclerosis (ALS), or spinal cord injury. BCIs convert brain electrical signals to commands to control external devices. Numerous research studies have been carried out to improve BCI technology over the past few decades [2,3].

EEG has important advantages that make it the first choice of physicians in emotion, epilepsy, sleep research, and clinical studies: high temporal resolution, ease of use, and affordability. Engineers use signal processing algorithms and machine learning approaches to interpret EEG signals. In recent years, lower-cost brain signal recording devices have been developed, the computational capabilities of computer systems have increased, and new machine learning techniques have been proposed [4]. Thus, brain research through BCIs has improved, and we have better knowledge about how the brain processes information than in previous years. However, there are other neuroimaging modalities—conventional methods mostly focused on scalp EEG measurements for understanding motor imagery (MI). In this study, we used inverse modeling and computed cortical level signals, and these signals were used to train classifiers. This research is important for noninvasive studies of the brain.

EEG signals recorded during MI are utilized in BCI studies. EEG source localization is mostly studied, especially on epileptic foci localization [5]. Various channel numbers are used in the recordings. EEG systems can have from a few electrodes to 256 electrodes. It has been said that source localization success decreases if the number of electrodes is low, especially when less than 32 [6]. In this study, we calculate EEG source imaging success for BCIs in the MI area. For low- and high-density channel numbers, accuracies were calculated. This study maps EEG signals to cortical regions using EEG source imaging. Thus, we eliminate the low spatial resolution of the EEG.

Using averaged signals in the cortical source space, CSP features are extracted using several selected Regions of Interest (ROIs). As noted by Fruitet and Clerc “It is difficult to distinguish phenomena that have close origins within the brain because the signals are measured with electrodes on the surface of the scalp and have crossed the dura, cerebrospinal fluid, and skull barriers. The solution we have focused on is to deconvolve EEG signals on the scalp by reconstructing cortical sources that are at the origin of the MI” [7]. Noninvasive EEG is susceptible to biologically and non-biologically caused artifacts because it measures tiny voltages from the scalp, reducing the quality of EEG signals. This experiment is conducted in mu and contains delta, theta, alpha, beta, and gamma attenuated EEG signals derived from a publicly available dataset.

However, it is hard to compute high-density EEG electrode data for better results since more channels result in a large amount of data. More electrodes provide more information; in this study, more and fewer electrode classification results are computed and compared. The hand, foot, and tongue take up a lot of space in the brain and can be seen on the homunculus, so these limbs are generally preferred. MI is one of the processes in which a subject thinks about moving a body part; its execution is performed mentally and does not use any muscle. It has been shown that MI activates the same brain regions as real movement [8]. When a person imagines performing a task or a limb movement without actually physically performing the movement, the neural system’s activation changes. Brain neuroimaging techniques have shown that similar areas of the brain are activated during MI and physical movement [9]. MI is a mental rehearsal in which actual movement is imagined without being performed as a motor function [8,10]. Although an MI task is only performed mentally, when a movement is imagined, similar areas of the brain are activated as when the actual movement is performed motorically [11].

In this study, we analyzed a dataset recorded using 118 channels. By reducing the number of electrodes, we achieved accuracy for 118 channels and a smaller number of channels. Using more channels gives better performance than fewer channels. Using fewer channels is not successful in epileptic foci localization studies [12]. It has been reported that using 118 channels helps improve source localization. That is why an epilepsy study is performed using high-density electrodes. Localization of epileptic foci is not accurate for less than 32 electrodes. In 2019, Michel and He reported that the sensitivity and specificity of Electrical Source Imaging (ESI) decreased significantly with a lower number of 32 electrodes and using a template head model [13]. The results parallel those for studies on epileptic foci localization; MI accuracy results are used in this study. The studies conducted by researchers are reviewed and the differences between this research and existing studies are highlighted. In the MI field, several EEG channel effects are observed.

Many studies have been carried out to develop EEG-based BCIs. Most of the studies deal with the performances obtained by the feature extraction operations on the electrodes placed on the scalp, followed by machine learning and deep learning classifications. Studies with EEG source imaging have also become widespread in recent years. While EEG has a temporal resolution that allows neural activity to be visualized in milliseconds, it can provide very little spatial information. In 2023, Dillen et al. [14] conducted a study involving 15 subjects and recorded MI using 64 electrodes. The study’s result demonstrated the feasibility of decoding MI from EEG data with a relatively limited number of sensors. In 2022, Abdullah et al. [15] studied EEG channel selection. Their research revealed that employing 10–30% of the total channels typically resulted in excellent performance, surpassing existing studies that use all available channels.

In 2021, Meng et al. [16] performed classification for the right hand and left hand with an Iterative Plot Convolutional Neural Network on EEG signals at the electrode level. In 2021, Singh et al. [17] published an extensive review paper on data collection, motor image training, signal preprocessing, feature extraction, channel and feature selection, classification, and performance measures in the motor image EEG field. In 2021, Zhang et al. [18] showed that the use of fewer EEG channels by channel selection using the automatic channel selection method not only reduces the computational complexity but also improves the classification performance in MI. In 2020, Saxena et al. [19] investigated functional brain activation by source localization for the right hand, left hand, two feet, and tongue and observed that the premotor cortex, primary motor cortex, postcentral gyrus, and posterior parietal cortex are significantly activated compared to other cortical areas of the brain. In 2017, Handiru et al. [20] achieved 10% higher success in arm movement by performing operations in the source space compared to channel-level signals. In a related study, 118 EEG channels were used, and operations were carried out in the source space to detect the movement of the right hand in four directions: north, south, east, and west. In 2020, Hou et al. [21] reported that they increased the classification success by extracting features from the signals in the patches they placed on the brain with EEG source localization and by classifying with convolutional neural networks. In 2004, Qin et al. [22] published a pilot study using source analysis. It was reported that the conversion of electrode EEG signals to source potentials helps to classify motor image EEG signals. In that study, one of the first studies in this field, data were recorded with 59 channels, including right hand and left hand motor imagination signals. Independent Component Analysis (ICA) and equivalent dipole analysis methods were used in the study. If the equivalent dipole occurs in the relevant brain lobe, it is accepted that the classification is correct. With this logic, an 80% classification accuracy was achieved. It has been reported that changes are observed around the C4 electrode in the left hand imagination and around the C3 electrode in the right hand imagination. It has been reported that the MI of the feet is around the Cz electrode. Because the areas are close, it is difficult to decide which foot the MI belongs to. In 2005, Kamousi et al. [23] reported promising results using source localization in right hand and left hand MI for four subjects.

From the literature review, it is apparent that the effect of the number of EEG electrodes on source localization epileptic foci localization has been investigated, but that the MI issue has not been studied so far. Motivated by this, we analyze the impact of employing a different number of EEG electrodes to the addressed issue. To the best of our knowledge, this paper’s notable contributions include analyzing the effect of the number of channels on cortical signals calculated from motor imaginary EEG recordings and exploring the Brodmann region combinations that will provide the highest performance.

## 2. Materials and Methods

**Figure 1:**
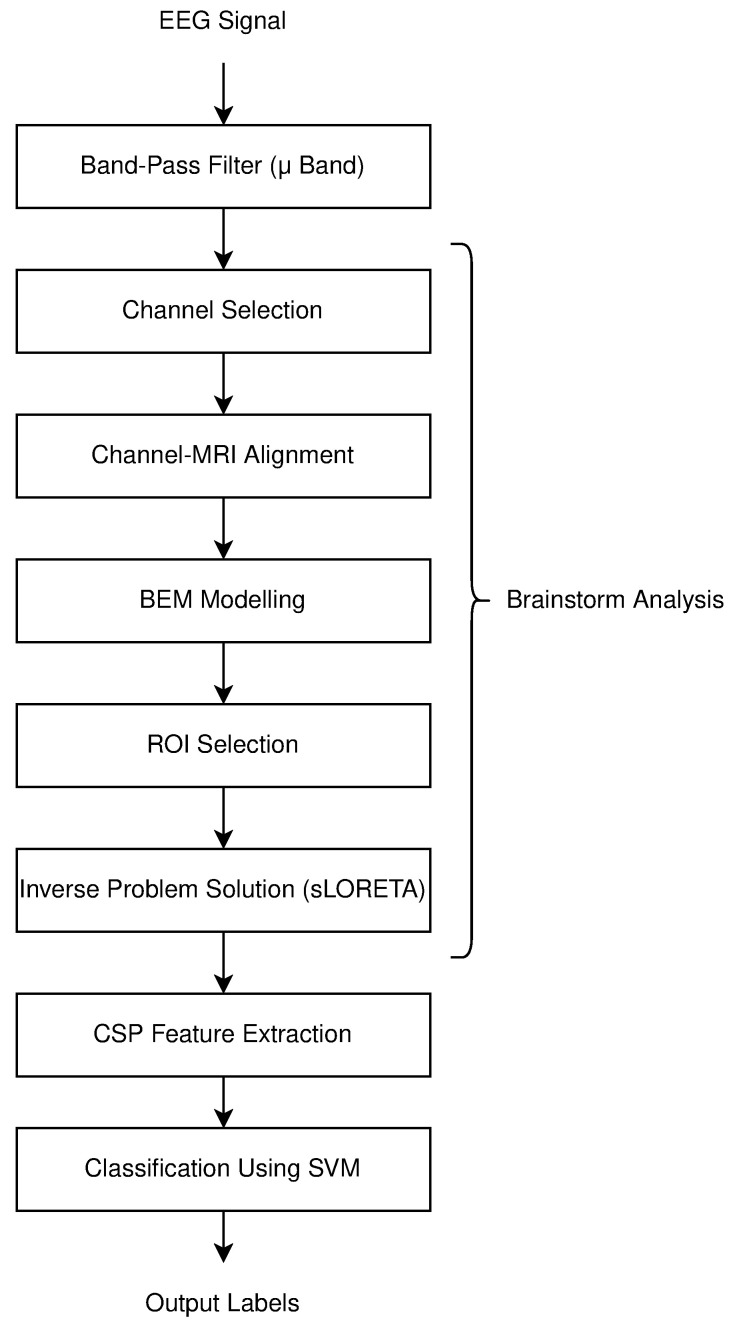
Flowchart of process steps in motor imagery classification.

The processes conducted in this study are depicted in the flow chart below in Figure 1. A summary of the methods used for feature extraction and classification applied to the existing EEG signals is provided. EEG signals are first filtered with a band-pass filter in the 8–13 Hz range, as significant changes in these rhythms occur during motor imagery. Next, the standard steps for addressing the inverse problem are performed using Brainstorm software (version 2019 and 2024). CSP feature extraction is applied to the processed signals, followed by classification using SVM machine learning. It is determined whether the motor imagery corresponds to the right hand or the right foot. During the results phase, outcomes are evaluated using five metrics. The findings obtained with different numbers of channels provide significant insights.

### 2.1. Dataset

The BCI Competition Dataset IVa for BCI Competition III is used in this study [24]. Data for five healthy subjects are included in this publicly available dataset. The right hand and right foot MI signals are classified. A total of 118 high-density EEG electrodes are used for recording the signals. Electrophysiological recordings are obtained using BrainAmp amplifiers along with a 128-channel Ag/AgCl electrode cap provided by ECI. The experiment involved recording 118 EEG channels according to the extended International 10–20 system. The signals underwent band-pass filtering between 0.05 Hz and 200 Hz and were digitized at a sampling rate of 1000 Hz with 16-bit resolution, resulting in a voltage accuracy of 0.1 μV. Additionally, analysis of the dataset was supported by the existence of a downsampled version at 100 Hz.

A total of 280 (train+test) signals were recorded. Subject names were coded using two letters for privacy. Training and testing numbers varied for each subject. Only the right hand and right foot signals were accessible for the competition. The number of training and test trials are listed in Table 1 for each subject in the dataset. Training and testing samples vary for each subject, and the total number of them is 280 for all subjects.

### 2.2. Signal Preprocessing

We used a 0.5–2.5 s interval for Dataset 4a and band filtered using 8–13 Hz for the mu band. Because EEG readings have low spatial resolution, EEG source localization is used to overcome this issue and increase classification accuracy. MI-EEG decoding is the most important part of biosignal processing. Conventionally, feature extraction and classification of EEG signals are made on the electrode level. ESI converts scalp signals to the brain cortical source area and faciliates source decoding of MI-EEG [25]. EEG data were processed using the sLORETA method [26] in the open-source software Brainstorm [27], and cortical signals were obtained and used for feature extraction and classification.

### 2.3. Brainstorm Workflow

#### 2.3.1. Number of Channels Selection

In clinical and BCI applications, studies where different numbers of electrodes are preferred are encountered. Scientists have put forward different opinions about how many electrodes should be used. Luck, in his 2014 [12] study, suggested that 32 electrodes may be sufficient for many experiments; he reported that with the use of more electrodes, some problems may be more difficult to detect and solve, and thus the quality of EEG data may decrease further. In 2002, Teplan [28] reported that the spatial resolution would increase with the use of a large number of up to 256 high-density recording electrodes. With the use of more electrodes in multiple EEG recordings, more time and effort will be required to place the electrodes. In addition, the analysis of the obtained records requires more processing and it is necessary to use expensive systems.

When EEG source analysis studies are examined, it has been demonstrated that a small number of electrodes may cause errors in the source analysis, and the use of at least 64 EEG electrodes can result in higher accuracy in source analysis [29,30,31,32,33].

Electroencephalogram (EEG) recordings can often be obtained with varying numbers of channels, but a standard EEG system usually has 19 channels. These channels consist of electrodes placed on the scalp to measure brain activity from different regions. These are recorded through electrodes placed in various positions to examine brain activity more precisely in targeted areas.

However, more channels can be used in special EEG recordings or research studies. EEG systems containing more channels can be used, especially for the examination of clinical conditions or neurological research. EEG recordings are particularly important in situations where different channels provide the opportunity to study specific areas of brain activity in greater detail.

**Figure 2:**
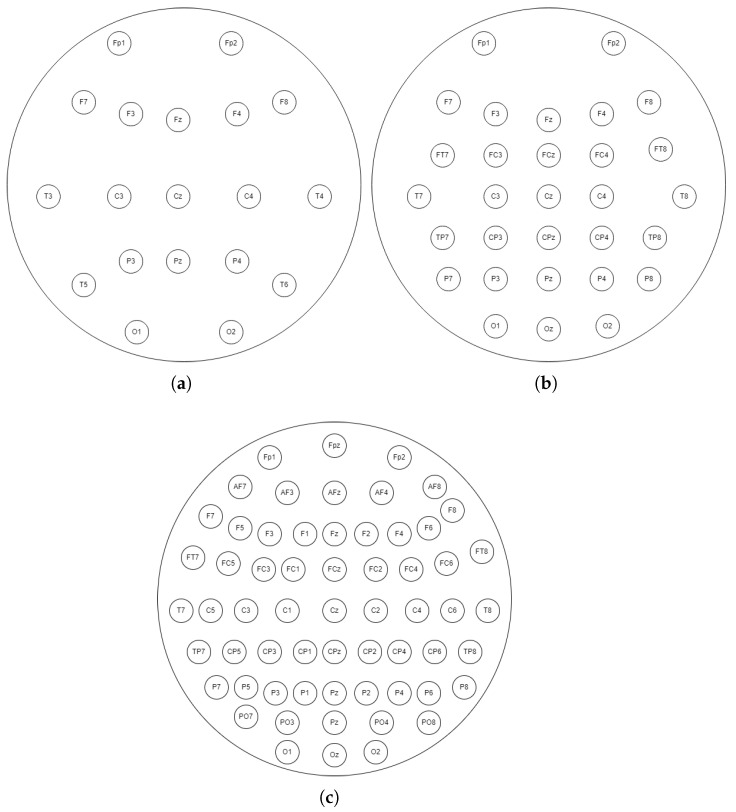
EEG 19-, 30-, and 61-channel caps derived from 118 channels in this study. (**a**) 19-channel EEG cap. (**b**) 30-channel EEG cap. (**c**) 61-channel EEG cap.

In this study, the effect of the number of electrodes on the correct classification of MI is examined. For this purpose, the accuracy values obtained using 118 electrodes and 19-, 30-, and 61-channel EEG signals derived from these EEG signals are obtained and compared separately. The number of electrodes and their positions for 19, 30, and 61 channels are given in Figure 2.

#### 2.3.2. Head Model

Researchers can use realistic head models or simplified spherical models to acquire source-level EEG signals. Also, individual MRI images can be used to improve results. The literature considers four layers of head models: brain, cerebrospinal fluid, skull, and scalp. In recent years, the widespread use of MRI technology and more powerful computer hardware to perform calculations have caused many studies to rely on realistic head models. The most commonly used methods are the Boundary Element Method (BEM), Finite Element Method (FEM), and Finite Difference Method (FDM). If there is a real MRI image of the patient or the subject who voluntarily participated in the experiment, creating a realistic head model from this image can be very useful for accuracy of the calculations. If there is no MRI image of a subject, then it will be more meaningful to use MRIs presented as templates in the literature. In this study, the default BEM head model in Brainstorm is used.

#### 2.3.3. Region of Interest

Cortical regions of interest occur on the sensorimotor area. They are called patches in Brainstorm. They reduce the dimension of data to be processed and help to extract valuable information about related MI tasks. According to Xygonakis et al. [34], 24 regions are defined by considering the Brodmann areas and other neuroanatomical structures.

**Figure 3:**
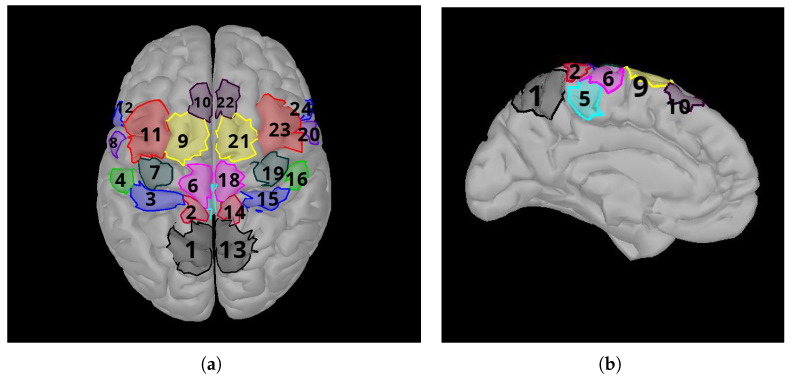
Brainstorm patch labels used in the study. (**a**) Top view of brain. (**b**) Left view of brain. Corresponding areas (1: Somatosensory Association Cortex (SAC), 2: Primary Foot Somatosensory Area (S1F), 3: Primary Hand Somatosensory Area (S1H), 4: Secondary Somatosensory Area (S2), 5: Cingulate Motor Area (CMA), 6: Primary Foot Motor Area (M1F), 7: Primary Hand Motor Area (M1H), 8: Primary Lip Motor Area (M1L), 9: Supplementary Motor Area (SMA), 10: Presupplementary Motor Area (pSMA), 11: Dorsal Premotor Cortex (PMd), 12: Ventral Premotor Cortex (PMv)). Regions numbered 13 to 24 are located in the right lobe. They are the right lobe counterparts of regions numbered 1 to 12, located in the left lobe, respectively.

These areas are the presupplementary motor area (pSMA), supplementary motor area (SMA), cingulate motor area (CMA), dorsal premotor cortex (PMd) and ventral premotor cortex (Pmv), primary foot motor area (M1F), primary hand motor area (M1H) and primary lip motor area (M1L), primary foot somatosensory area (S1F), primary hand somatosensory area (S1H), secondary somatosensory area (S2), and the somatosensory association cortex (SAC). As motor imageries of the right hand and right foot are used, the most suitable brain regions are selected for the calculations. Only the patchs’ source time series are used for feature extraction and classification. The brain lobes corresponding to the right hand and right foot are the $M1H_{L}$ and $M1F_{L}$ regions, which are the primary areas responsible for motor movement. All possible combinations of these regions are included. A total of six regions’ combinations are used for the classifications. For right hand and right foot MI classification, accuracy is calculated using the $M1H_{L}$ and $M1F_{L}$ regions in all combinations, and quadruple combinations of the remaining 16 regions are added and calculated 1820 times. The best accuracy results are summarized in the table. Area 17, CMA, is not on the surface of the brain and cannot be seen from the outside because it is located in the interior. Area 17 is its counterpart of area 5 in the right lobe. The Brodmann areas are given in Figure 3.

#### 2.3.4. Electrode to Source Signal (Inverse) Modeling

In this study, the processing steps include preprocessing, feature extraction, and classification. Training and test data are used as given in the dataset. First, some of the recordings are used to determine which part of the signal should be processed. It is decided to use a 0.5–2.5 s time interval. Source potentials are obtained using the inverse problem solutions of the Brainstorm software. Then, the CSP features are extracted and classified using SVM.

### 2.4. Feature Extraction

CSPs are used for feature extraction. CSP, first proposed to discriminate 2 class problems, is widely used in EEG analysis. Three filter pairs are used for the best results. CSP, mostly used in EEG MI, is used in this study. It is a spatial filter based on supervised learning. It is widely used in the literature because it has high accuracy and the calculations needed are simple. This method maximizes one class’s variance while minimizing the other class’s [35,36]. One EEG epoch’s normalized spatial covariance matrix is calculated as follows:

Here, *D* shows the C×S matrix trial. *C* shows the number of channels, and *S* is the number of samples. The trace is the $DD_{t}$ multiplication sum of the diagonal elements. The spatial covariance matrix is calculated by averaging each class trial.

(1) $$M=\frac{DD^{t}}{trace(DD^{t})}$$

Two matrices are generated—one for class 1 and for class 2. The covariance matrix is summed, and the combined covariance matrix is calculated as follows:

(2) $$M_{c}=\bar{M_{1}}+\bar{M_{2}}$$

Expression $M_{c}$ is decomposed into eigenvectors as

(3) $$M_{c}=E_{c}\lambda_{c}E_{C}^{T}$$

Here, $E_{c}$ is the matrix of eigenvectors, and $\lambda_{c}$ is the diagonal matrix of eigenvalues. Then, the whitening transformation is used to equalize the variance values in eigenspace.

(4) $$W=\sqrt{\lambda_{C}^{-}1}E_{C}^{T}$$

*W* is employed to transform the average covariance matrices into

(5) $$K_{1}=W\bar{M_{1}}W^{T}and\;K_{2}=W\bar{M_{2}}W^{T}$$

In this case, *K*_1_ and *K*_2_ have shared eigenvectors, and the total of their corresponding eigenvalues are consistently equal to 1, thus ensuring that

(6) $$K_{1}=U\lambda_{1}U^{T},K_{2}=U\lambda_{2}U^{T}\lambda_{1}+\lambda_{2}=I$$

Here, *I* represents the identity matrix. Lastly, the projection matrix *P* is derived from the CSPs and can be interpreted as vectors representing the time-invariant EEG source distribution vectors. Using this projection matrix, the decomposition of a trial *D* is performed as follows:

(7) Z=PD

As the sum of the corresponding eigenvalues is consistently equal to one, the variances of the initial and final rows in *Z* serve as appropriate features for classification. In this study, we employed these variances from the first and last rows as our feature set. Their calculation method is described as follows:

(8) $$V=\frac{\sum {\left(Z_{R}-\bar{Z_{R}}\right)}^{2}}{L-1}$$

Here, *Z* represents a row within the matrix *Z*, and *L* signifies the length of this particular row.

### 2.5. Classification

This work utilizes SVM, a well-known kernel-based classifier for classification. This method was introduced by Boser et al. [37] in 1992. SVM assigns new examples to the corresponding classes. If the binary class problem is used to train SVM, it produces the best hyperplane to separate the two classes. The goal is to draw the farthest line between the two classes. Although this classifier was developed for the binary class problem, new approaches have been added for cases with more than two classes. It can also classify nonlinearly separable classes. SVM is easy to apply to classification problems. These classifiers do not take input parameters. There is no prior knowledge or assumptions made about the distribution of data in space. The input and output values are processed on the training set. Thus, decision functions are obtained. After training, inputs are associated to corresponding outputs for testing.

## 3. Results

As a novel contribution to the literature, this study seeks to answer whether there is a relationship between the number of channels and the classification performance for MI.

EEG data are compared by performing feature extraction and classification with MATLAB R2017b and R2024b using backward problem-solving for 19, 30, 61, and 118 channels with the Brainstorm software.

We use four different configurations in the experiments. The channels are selected according to the most popular commercial EEG caps in the literature. The SVM method with linear and radial basis function (RBF) kernels is used to perform the experiments. The results based on metrics such as accuracy, sensitivity, specificity, precision, and F1-score are presented in Table 2, Table 3, Table 4 and Table 5. In our calculations, when right hand images are correctly recognized, the true positive (TP) value increases, while if they are incorrectly classified as right foot images, the false negative (FN) value increases. Similarly, correctly identifying right foot imageries increases the true negative (TN) value, while misclassifying them as right hand imageries increases the false positive (FP) value.

When Table 1 is examined, the number of samples in the training and testing datasets varies for each subject. The results for 1820 combinations are evaluated and shown for the combination with the highest accuracy.

In Table 2, it is seen that the “al” subject achieved 100% success because it has 224 training samples. The subject’s samples are far more than the others and as a result, its success is also higher than the other subjects.

In Table 3, the number of channels increased from 19 to 30. When the average results are taken into account, it can be observed that 30 channels are more successful than the 19-channel case.

The results are given for 61 channels in Table 4. When the results of 61 and 30 channels are compared, generally, slightly improved results are obtained with 61 channels than with 30 channels.

In Table 5, the number of channels increased from 61 to 118. The evaluation of the results reveals that the accuracy and F1-scores in the 61-channel measurements are higher than in the 118-channel measurements. In the method proposed here, only the specificity and sensitivity values increase when 118 channels are used. This shows that 61 channels provide better results than 118-channel measurements.

When examining the tables, accuracy improves to a certain extent as the number of channels increases. The main improvement is seen in the precision and specificity values. Considering the results, fewer channels suffice for non-critical applications, 61 channels are meaningful for accuracy-critical applications, and high-density channel usage is essential for applications requiring greater specificity and precision values.

The averages of the results in Table 2, Table 3, Table 4 and Table 5 are calculated, and the average accuracy and F1-score values obtained for different channel numbers are presented in Table 6.

**Figure 4:**
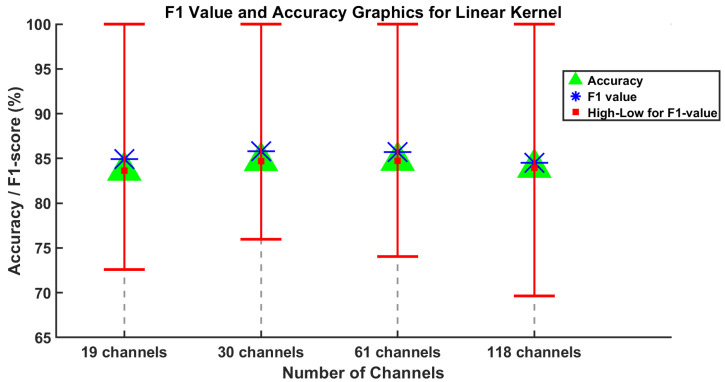
Results of the SVM method using linear kernel are obtained for the average values of F1-score and accuracy evaluation metrics for channel numbers 19, 30, 61, and 118.

**Figure 5:**
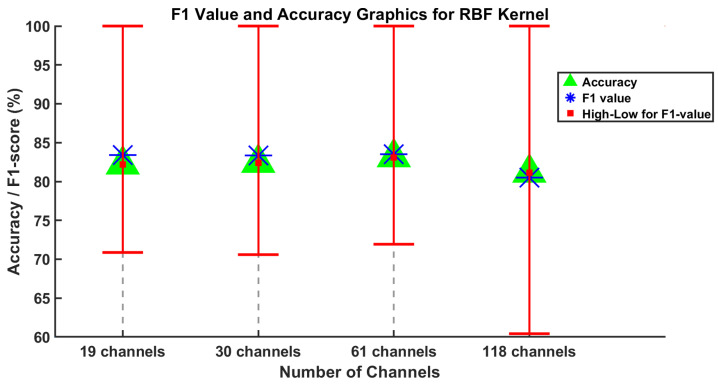
Results of the SVM method using RBF kernel are obtained for the average values of F1-score and accuracy evaluation metrics for channel numbers 19, 30, 61, and 118.

Figure 4 and Figure 5 display the average F1-score, average accuracy, and the lowest and highest F1-score values for five subjects across varying channel numbers. Considering all the channel numbers illustrated in Figure 4, the best results using the proposed SVM method with linear kernel are achieved with 30 and 61 channels. Furthermore, similar results are observed using the SVM method with the RBF kernel, as shown in Figure 5. In this context, the accuracy values from 19 and 118 channels can be disregarded as they are somewhat lower than those from 30 and 61 channels.

## 4. Discussion

In this study, we aimed to answer whether there is a relationship between the number of EEG channels and the classification performance of MI. Four different numbers of EEG channel configurations are selected. The main goal of the analysis is to determine the positive or negative relationship between MI and the number of channels. Many studies have investigated the number of channels that should be used, especially in epilepsy studies [38]. This study aimed to determine whether and to what extent higher-density EEG provides additional useful information. A 118-channel dataset from five healthy individuals was used, and three subsets with 19, 30, and 61 channels were created from it. The results indicated that increasing the number of channels increased the prediction accuracy to a certain level, but the success rate decreased slightly when increasing from 61 to 118 channels. Despite the reduction in the represented area, fewer channels still produced comparable results.

Various reports can be found in the literature regarding the effect of an increase in the number of channels. Some studies have indicated that increasing the number of EEG channels increases the success, while others have reported that no significant change or a decrease in success is also possible. For example, in 2017, Schirrmeister et al. [39] demonstrated that the data from more channels improves classification accuracy. They specifically employed deep learning models to show how more channels improve classification performance. Similarly, in 2019, Roy et al. [40] reported that the data from more channels helps algorithms filter noise more effectively and reduce the impact of erroneous data. In 2018, Lotte et al. [41] found that some channels carry less information and using these channels can degrade classification performance.

Furthermore, in 2022 Soler et al. [42] showed that optimal subsets with six electrodes achieved equal or higher accuracy than 200+ channel HD EEG. In 2021, Zhang et al. [43] reported that increasing the number of EEG channels does not always improve classification performance and can sometimes degrade it. According to their experiments, certain classification algorithms performed better with 32-channel EEG data than 64-channel data. In 2024, Ajra et al. [44] reported that successful classification could be achieved even with single-channel EEG classification, which is important for the use of single-channel EEG in real-world applications.

EEG systems vary in channel count from 1 or 8 channels to 19, 32, and 64–256 (HD-EEG) channel configurations. HD-EEG provides detailed observation of brain activity and is used in both healthy and clinical populations. It has been shown that increasing the number of channels can increase the spatial resolution and classification accuracy. Electroencephalogram (EEG) caps with fewer channels are less expensive, more straightforward to install, and can reduce noise and data redundancy. They also result in lower computational costs and higher accuracy. However, the reduction in channels can potentially lead to low spatial resolution, information loss, while the complexity of patterns may be underestimated, and classification accuracies may be diminished when the number of channels is insufficient.

Ketola et al. [45] reported that high sampling rates and the use of large numbers of electrodes cause the data size to increase rapidly. Suwannarat et al. [46] stated that the use of a small number of EEG channels is especially important for areas such as wearable solutions in healthcare and mobile rehabilitation. Shiam et al. [47] emphasized that by making an effective channel selection and classifying after removing unnecessary information, accuracy increases.

Most multi-channel EEG systems are not portable and rely on fixed hardware. Therefore, it is crucial to find an optimal balance between the number of channels and system usability; this is particularly important in the context of application-specific solutions.

In this study, four distinct channel configurations were selected for experiments to assess the validity of employing the effects of different channels. Four different subsets of EEG channels were used to solve the inverse problem and were compared according to accuracy and various measures of success in EEG MI. The results in this study show that even with fewer channels, it is possible to obtain results comparable to those obtained with multiple channels. Although multichannel EEG expands the representation space, it did not raise the predicted learning level as expected, and no significant increases were observed.

The variation in classification performance concerning the number of EEG channels is influenced by both neurophysiological and computational factors. Using a small number of channels, such as 19, limits the spatial resolution and restricts accurate source localization. Conversely, using too many channels, such as 118, may increase model complexity and risk overfitting due to redundant information and noise. A moderate range of 30 to 61 channels generally provides sufficient spatial coverage and a balanced signal-to-noise ratio, thereby supporting more reliable classification after source localization.

It can be concluded that increasing the number of electrodes does not always enhance classification performance. To achieve the best results, the number and placement of electrodes should be carefully determined, considering the classification algorithms and data processing techniques utilized. Statistically, a larger number of subjects can lead to better results.

Increasing the number of EEG channels generates more data, increases computation time, and complicates data processing and analysis. Using fewer channels can reduce costs and setup time and may have the advantage of increasing patient comfort. Each additional channel increases signal processing time and data size, which increases cost and complexity. Minimizing the number of EEG channels in research is beneficial as it saves computation time and cost.

## 5. Conclusions

This study investigates the effect of the number of channels used on the classification of EEG signals following EEG source imaging. It is observed that the performance is good when all channels in the dataset are used, but higher performance is obtained when 30 and 61 channels are used. The accuracy of using 19 channels and 118 channels are close to each other.

The system is trained and tested using EEG potentials obtained with sLORETA. The 19-channel results show less success, but there is no significant drop. The 30-channel and 61-channel results are better than those obtained with 19 channels. The 118-channel results are better than those for 19 channels but less than for 30 and 61 channels. The best results are achieved when 61 channels are utilized.

One shortcoming of the current study is the quite limited number of subjects. The use of EEG data from only five subjects implies important limitations that may affect the reliability and generalizability of the findings. With such a small sample size, the dataset may not fully capture interindividual variability, increasing the risk of overfitting, especially in machine learning applications. The statistical power is also limited. Therefore, findings based on this dataset should be interpreted with caution and should be considered preliminary. It would be of value to provide results with more subjects in the future. However, the results reported in this work were obtained using all the channels on the cap without any channel selection and were compared with a standard EEG caps configuration.

Future work should aim to improve the generalizability of this method by testing the source localization algorithms, feature extraction, and classification on larger datasets and more subjects. A larger number of channels would enable more detailed and precise measurements but can also lead to more complex data processing and analysis. On the other hand, systems with fewer channels are less costly, more portable, and easier to use.

## Figures and Tables

**Table 1:** Number of training and test trials for each subject.

Subject	Number of Training Samples	Number of Test Samples
aa	168	112
al	224	56
av	84	196
aw	56	224
ay	28	252

**Table 2:** Performance comparison between linear and RBF kernels for each subject for 19 channels.

Linear						RBF				
**Subject**	**Acc**	**Sen**	**Spec**	**Pre**	**F1**	**Acc**	**Sen**	**Spec**	**Pre**	**F1**
aa	66.96	81.67	50	65.33	72.59	66.96	75	57.69	67.16	70.87
al	100	100	100	100	100	100	100	100	100	100
av	74.49	78.57	70.41	72.64	75.49	72.45	80.61	64.29	69.30	74.53
aw	83.04	83.64	82.46	82.14	82.88	82.59	80.91	84.21	83.18	82.03
ay	93.65	97.54	90.00	90.15	93.70	88.89	97.54	80.77	82.64	89.47
Mean	83.63	88.28	78.57	82.05	84.93	82.18	86.81	77.39	80.46	83.38

The table reports accuracy (Acc), sensitivity (Sen), specificity (Spec), precision (Pre), and F1-score (F1) for each subject using linear and RBF kernels.

**Table 3:** Performance comparison between linear and RBF kernels for each subject for 30 channels.

Linear						RBF				
**Subject**	**Acc**	**Sen**	**Spec**	**Pre**	**F1**	**Acc**	**Sen**	**Spec**	**Pre**	**F1**
aa	72.32	81.67	61.54	71.01	75.97	68.75	70.00	67.31	71.19	70.59
al	100	100	100	100	100	100	100	100	100	100
av	74.49	83.67	65.31	70.69	76.64	72.45	82.65	62.24	68.64	75.00
aw	83.04	83.64	82.46	82.14	82.88	83.48	83.64	83.33	82.88	83.26
ay	93.65	93.44	93.85	93.44	93.44	87.30	95.90	79.23	81.25	87.97
Mean	84.70	88.48	80.63	83.46	85.79	82.40	86.44	78.42	80.79	83.36

**Table 4:** Performance comparison between linear and RBF kernels for each subject for 61 channels.

Linear						RBF				
**Subject**	**Acc**	**Sen**	**Spec**	**Pre**	**F1**	**Acc**	**Sen**	**Spec**	**Pre**	**F1**
aa	72.32	70.00	75.00	76.36	74.04	71.43	68.33	75.00	75.93	71.93
al	100	100	100	100	100	100	100	100	100	100
av	71.43	84.69	58.16	66.94	74.77	70.92	73.47	68.37	69.90	71.64
aw	87.05	86.36	87.72	87.16	86.76	87.50	85.45	89.47	88.68	87.04
ay	92.86	96.72	89.23	89.39	92.91	85.71	99.18	73.08	77.56	87.05
Mean	84.73	87.55	82.02	83.97	85.70	83.11	85.29	81.18	82.41	83.53

**Table 5:** Performance comparison between linear and RBF kernels for each subject for 118 channels.

Linear						RBF				
**Subject**	**Acc**	**Sen**	**Spec**	**Pre**	**F1**	**Acc**	**Sen**	**Spec**	**Pre**	**F1**
aa	69.64	65.00	75.00	75.00	69.64	66.07	48.33	86.54	80.56	60.42
al	100	100	100	100	100	100	100	100	100	100
av	68.37	80.61	56.12	64.75	71.82	66.84	70.41	63.27	65.71	67.98
aw	89.29	86.36	92.11	91.35	88.79	90.18	89.09	91.23	90.74	89.91
ay	92.46	93.44	91.54	91.20	92.31	82.54	95.90	70.00	75.00	84.17
Mean	83.95	85.08	82.95	84.46	84.51	81.13	80.75	82.21	82.40	80.50

**Table 6:** Comparison of average accuracy and average F1-score according to different channel numbers and SVM kernel types.

Number of Channel	Kernel	Acc (%)	F1-Score (%)
19-channel	Linear	83.63	84.93
19-channel	RBF	82.18	83.38
30-channel	Linear	84.70	85.79
30-channel	RBF	82.40	83.36
61-channel	Linear	84.73	85.70
61-channel	RBF	83.11	83.53
118-channel	Linear	83.95	84.51
118-channel	RBF	81.13	80.50

## Data Availability

The data utilized in this study are from Data Set IVa of the BCI Competition III. This dataset is publicly available for academic and research purposes and can be accessed through the official BCI Competition website at http://www.bbci.de/competition/iii/ (accessed on 17 February 2024).

## Abbreviations

The following abbreviations are used in this manuscript:

Acc	Accuracy
ALS	Amyotrophic Lateral Sclerosis
BCI	Brain–Computer Interface
BEM	Boundary Element Method
CSP	Common Spatial Patterns
CMA	Cingulate Motor Area
EEG	Electroencephalography
ESI	Electrical Source Imaging
F1	F1-score
FDM	Finite Difference Method
FEM	Finite Element Method
HD-EEG	High-density EEG
ICA	Independent Component Analysis
MI	Motor Imagery
ML	Machine Learning
M1F	Primary Foot Motor Area
M1H	Primary Hand Motor Area
M1L	Primary Lip Motor Area
MRI	Magnetic Resonance Imaging
PMd	Dorsal Premotor Cortex
PMv	Ventral Premotor Cortex
Pre	Precision
pSMA	Presupplementary Motor Area
RBF	Radial Basis Function
ROI	Region of Interest
Sen	Sensitivity
S1F	Primary Foot Somatosensory Area
S1H	Primary Hand Somatosensory Area
S2	Secondary Somatosensory Area
SAC	Somatosensory Association Cortex
sLORETA	Standardized Low-Resolution Electromagnetic Tomographic Analysis
SMA	Supplementary Motor Area
Spec	Specificity
SVM	Support Vector Machine
